# Trio-based exome sequencing and high-resolution HLA typing in families of patients with autoimmune adrenal insufficiency and autoimmune polyglandular syndrome

**DOI:** 10.1371/journal.pone.0312335

**Published:** 2024-10-18

**Authors:** Anastasiia Buianova, Marina Yukina, Valery Cheranev, Oleg Suchalko, Anna Shmitko, Alina Samitova, Nurana Nuralieva, Elena Kulagina, Elena Savvateeva, Ekaterina Troshina, Denis Rebrikov, Dmitry Gryadunov, Dmitriy Korostin

**Affiliations:** 1 Center for Precision Genome Editing and Genetic Technologies for Biomedicine, Pirogov Russian National Research Medical University, Moscow, Russia; 2 Endocrinology Research Centre, Ministry of Health of Russia, Moscow, Russia; 3 Abu Dhabi Stem Cells Center, Abu Dhabi, United Arab Emirates; 4 Engelhardt Institute of Molecular Biology (EIMB), Center for Precision Genome Editing and Genetic Technologies for Biomedicine, Russian Academy of Sciences, Moscow, Russia; ICMR-National Institute for Research in Tuberculosis, INDIA

## Abstract

Autoimmune adrenal insufficiency (AAI) is a rare disease. This research evaluates three patients with AAI, including autoimmune polyglandular syndrome (APS) type 2. Two patients had APS or AAI during childhood, and one had a history of endocrine autoimmune disease, indicating a possible hereditary basis of the condition. Trio-based exome sequencing and high-resolution HLA typing were employed to analyze patients and their parents. Benign or likely benign variants of the *AIRE* gene were identified in all participants of the study. These variants, coupled with clinical data and the results of antibody studies to type I interferons, helped to exclude APS-1. Patients with APS-2, in contrast to patient with AAI, inherited distinct variants of unknown significance in the *CLEC16A* gene, which is associated with autoimmune diseases, including AAI. Various risk alleles in other genes associated with autoimmunity were identified in all patients. HLA typing of class II loci revealed alleles related to APS. Nevertheless, the frequencies of the haplotypes identified are substantial in the healthy Russian population. Immunological tests can detect antibody carriers and assess the risk of autoimmune disease development. In the future, to identify genetic predictors of autoimmune endocrinopathies, it is recommended to analyze the whole genome of patients and their relatives, examining clinically relevant variants in non-coding regions.

## Introduction

Polyautoimmunity, a combination of multiple autoimmune diseases within a single patient, is frequently encountered in clinical settings [1,2]. Autoimmune polyglandular syndrome (APS) refers to the damage of two or more endocrine glands, leading to their failure, often accompanied by organ-specific non-endocrine autoimmune diseases [3,4]. There are two primary types of APS: 1 and 2. APS-1 is a disease caused by a mutation in the autoimmune regulator (*AIRE*) gene. It is usually inherited in an autosomal recessive manner. The syndrome comprises of three primary elements: autoimmune adrenal insufficiency (AAI), hypoparathyroidism, and mucocutaneous candidiasis. APS-1 typically appears during childhood [3,4]. It is important to note that APS-1 is one of the few types of monogenic autoimmune diseases that primarily affect the endocrine system and follow the principles of Mendelian inheritance [1,5]. Much more frequently, there is a complicated interplay between various genetic factors that determine susceptibility to illness and the environment [1,4,6]. A prime example is APS-2, also known as adult APS. This condition is typically characterized by the co-occurrence of features, namely AAI, type 1 diabetes mellitus (DM1), and/or autoimmune thyroid diseases such as autoimmune thyroiditis (AIT) or Graves’ disease (GD) [7].

Considering the polyautoimmunity phenomenon, it cannot be ruled out that impaired immune tolerance to self-antigens may be a common pathogenetic mechanism underlying the development of various autoimmune diseases. Studies supporting this hypothesis have identified several genetic loci crucial for regulating immunity and contributing to the predisposition to multiple pathologies at the same time [4,8,9]. Thus, certain alleles of the genes in the major histocompatibility complex (MHC) system have been linked to an increased risk of developing autoimmune diseases. Specifically, components of the human leukocyte antigens (HLA) class II encoded HLA-DRB1*03-DQA1*0501-DQB1*0201 and DRB1*04-DQA1*0301-DQB1*0302 haplotypes have been associated with various autoimmune diseases, including AAI [10,11]. These haplotypes have a high frequency in the general population, with around 20% occurring in central Italy [12]. Nevertheless, only a minority of carriers develops AAI.

Variations in the *PTPN22*, *CTLA-4*, *CIITA*, and other genes are believed to modulate the risk determined by HLA class II genes [9,13–15]. Most likely, the development of APS-2 components is determined by the combined effects of mutations in multiple genes that have clinically significant mutual influence. This necessitates a comprehensive investigation of multiple genes using exome sequencing, followed by the creation of a genetic roadmap. Accumulating data on correlations between phenotype and genotype will enable a clear determination of disease prognosis, as well as the appropriate tactics for subsequent evaluation, treatment, and monitoring. Additionally, a whole-exome analysis of the probands and their parents provides an opportunity to identify novel predictors of autoimmune diseases. This study examines three patients: two with APS-2 (Patient A and Patient B) and one with AAI (Patient C). Patient A and C developed APS or AAI in childhood, while patient B had a history of endocrine autoimmune disease. Assuming that autoimmune dysfunction in various organs and tissues in APS-2 stems from a common pathogenetic mechanism, we initially selected patients with the most severe manifestation of APS–AAI. As this is a preliminary investigation, additional patients with other manifestations will be included in future phases of the study. Our clinical findings suggest a possible hereditary basis of the disease. The article reports the results of genetic and immunological assessments conducted on these patients and their families.

## Materials and methods

### Patients

Patient A (female) was enrolled in the study at the age of 22 years. At the age of 16, she received a diagnosis of primary hypothyroidism due to autoimmune thyroiditis and has been taking levothyroxine sodium. By the age of 18, the patient exhibited clinical indications of primary adrenal insufficiency, including skin darkening around the knee and elbow joints as well as groin folds, vomiting, a craving for salty food, and weight loss of 16 kg. The symptoms progressed over time, and within two years, the patient experienced an Addisonian crisis, requiring urgent hospitalization. Hydrocortisone and fludrocortisone therapy was initiated during hospitalization. The patient presented with comorbidities, including Sutton nevus and latent iron deficiency. The patient’s mother experienced an acute cerebral circulatory disorder at the age of 50, while her father had chronic pancreatitis. Her maternal grandmother was diagnosed with nodular goiter, and her maternal grandfather had prostate cancer that metastasized to the stomach. Additionally, her paternal grandfather had arterial hypertension from a young age. The results of laboratory tests from the most recent hospitalization are shown in Table 1.

**Table 1:** Laboratory examination of patients upon the latest hospitalization.

Analysis	Indicator, reference range	Patient A	Patient B	Patient C
Biochemical blood test	Ca total, 2.15–2.55 mmol/L; Ca ionized, 1.03–1.29 mmol/L	Ca*, 2.29 mmol/L	Ca*, 2.4 mmol/L	Ca*, 2.51 mmol/L
	R, 0.74–1.52 mmol/L	1.46 mmol/L	1.28 mmol/L	1.36 mmol/L
	glucose, 3.1–6.1 mmol/L	4.64 mmol/L	4.63 mmol/L	4.68 mmol/L
	ALT, 0–55.0 U/L	10 U/L	16 U/L	10 U/L
	AST, 5.0–34.0 U/L	15 U/L	15 U/L	14 U/L
	creatinine, 50–98 μmol/L	61.6 μmol/L	66.5 μmol/L	84.3 μmol/L
	vitamin B12, 191–663 pg/mL	187 pg/mL	764 pg/mL	-
Thyrotropine	0.25–3.5 mIU/L	1.755 mIU/L (while undergoing levothyroxine sodium replacement therapy)	1.163 mIU/L	1.264 mIU/L
LH, FSH, estradiol	LH, 2.6–12.1 U/L	7.71 U/L	-	7.43 U/L
	FSH, 1.9–11.7 U/L	3.79 U/L	-	3.84 U/L
	estradiol, 97–592 pmol/L	150.43 pmol/L	-	166.09 pmol/L
Aldosterone, renin	aldosterone, 69.8–1085.8 pmol/L	-	78.3 pmol/L	-
	renin, 2.8–39.9 mU/L	33.21 mU/L	> 500 mU/L	24 mU/L
ACTH, cortisol	ACTH, 7.2–63.3 pg/mL	-	>2000 pg/mL	-
	cortisol during insulin hypoglycemia test, more than 500 nmol/L	-	peak cortisol level during insulin hypoglycemia test 126.7 nmol/L	-
Glycated hemoglobin	up to 6%	5.6%	5%	5.3%
Insulin, C-peptide	2.6–24.9 μU/mL	12.74 μU/mL	11.75 μU/mL	-
	1.1–4.4 ng/mL	2.98 ng/mL	2.11 ng/mL	2.6 ng/mL

Notes: Ca, calcium; Ca*, calcium, corrected for albumin; R, reticulocytes; ALT, alanine aminotransferase; LH, luteinizing hormone; FSH, follicle-stimulating hormone; ACTH, adrenocorticotropic hormone.

Patient B, a female participant, was enrolled in the study at the age of 28, presenting with complaints of skin darkening, dry skin, hair loss, general weakness, nausea, and cravings for salty foods. Primary autoimmune-related adrenal insufficiency was confirmed at the Endocrinology Research Center, Ministry of Health of Russia, through the insulin hypoglycemia test (Table 1). Administration of hydrocortisone and fludrocortisone commenced as treatment. The patient reported no menstrual disorders. Related pathologies included autoimmune thyroiditis in the euthyroid stage, nodular goiter (EUTIRADS 2), peptic ulcer of the duodenum, hemorrhoids, and a surgically removed lipoma on the back at age 28 years old. The patient was examined by a dermatologist due to suspicion of papillary lichen planus. The family history includes peptic ulcer disease in both the patient’s father and grandfather, as well as diffuse toxic goiter in the patient’s mother.

The female patient C underwent her initial examination at the Endocrinology Research Center, Moscow, Russia, at the age of 20. Based on the medical documentation provided, acute hypoxia was observed upon birth. Thereafter, the infant experienced diarrhea and weight loss from the first few days of life, with a diagnosis of anus atresia and intermediate fistula (surgical treatment was performed at 3.5 years old). Since the first year of life, the patient has experienced nausea, vomiting, diarrhea (unrelated to meals), and frequent fainting, particularly in connection with acute respiratory viral infections and emotional stress. As a result, she has been frequently hospitalized in the infectious diseases department. At age 15, she underwent her first hormonal examination due to a sudden 10kg weight loss, salt cravings, and skin darkening. Adrenocorticotropic hormone (ACTH) levels were measured at 1008 pg/mL. Blood cortisol levels were found to be 40.2 nmol/L, while aldosterone levels were 49 pmol/L. Renin levels were determined to be 141 IU/L. Primary adrenal insufficiency was diagnosed, and treatment with hydrocortisone and fludrocortisone was initiated. According to the patient’s medical history, reactive hepatitis was diagnosed at the age of 18, and ophthalmologic examination revealed partial ptosis of both eyelids. Table 1 presents the laboratory test results from the patient’s most recent hospitalization. The examination revealed the following comorbidities: hyperprolactinemia without a pituitary adenoma (the patient was started on treatment with a dopaminergic receptor agonist) and moderate regurgitation due to mitral valve anterior leaflet prolapse. It is noteworthy that the patient was additionally diagnosed with esophageal candidiasis. The patient’s medical history indicates several hereditary conditions. Her mother’s medical records indicate diagnoses of ovarian cancer, primary hypothyroidism resulting from autoimmune thyroiditis, nodular goiter, and polycystic ovary syndrome. Additionally, other family members have been diagnosed with breast cancer (maternal grandmother), gastric cancer (maternal grandmother’s father), rheumatoid arthritis (maternal grandfather), and lung cancer (paternal grandfather).

**Fig 1:**
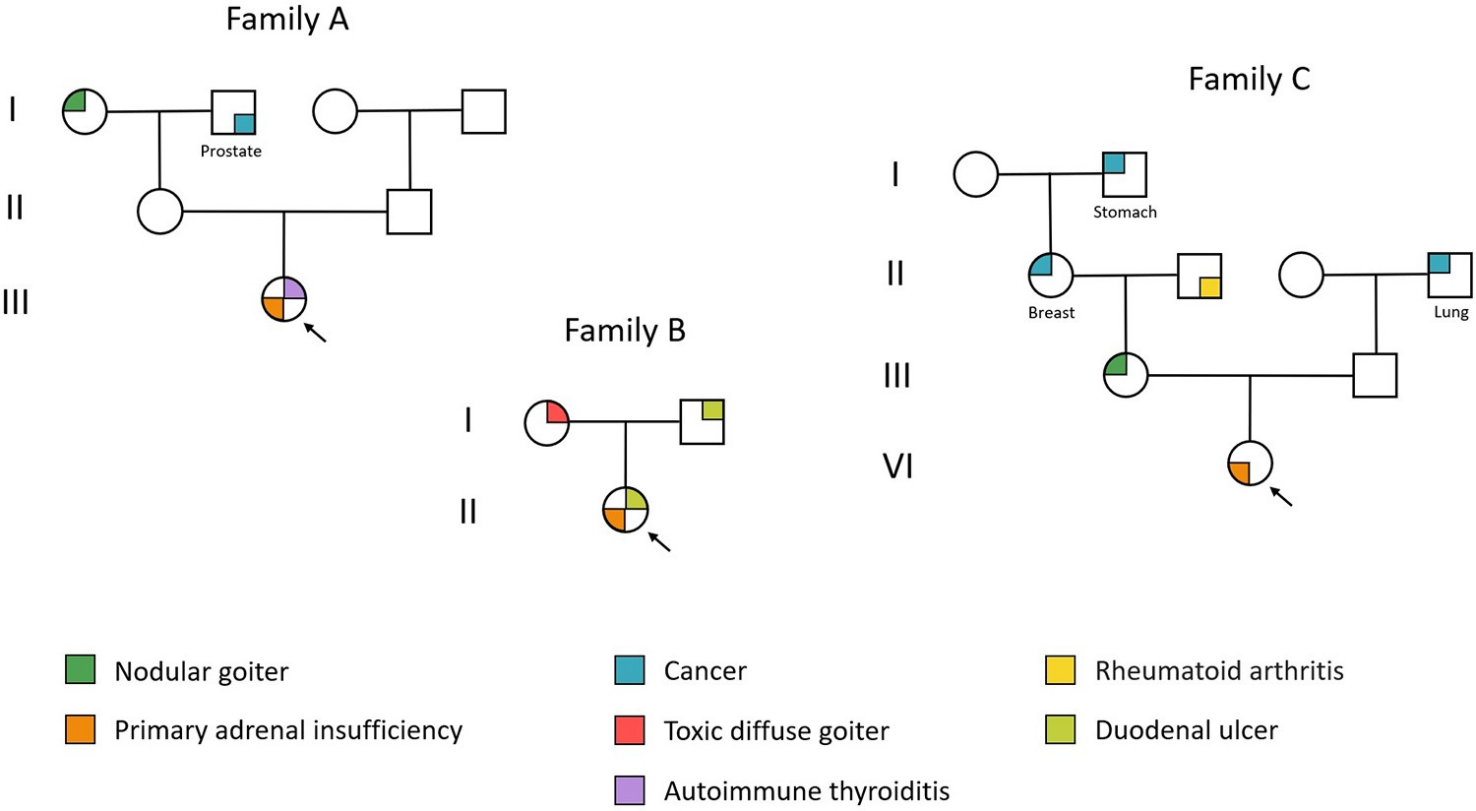
Graph of three families’ pedigrees displaying the hereditary traits of the probands. Only family members with reliable health information are included. The pedigrees do not show common multifactorial diseases, such as hypertension, or diseases with an unclear link to autoimmunity, starting from the grandparents of the probands’ generation and earlier. Healthy siblings are not represented.

The probands’ hereditary history is shown in Fig 1.

This study was conducted according to the guidelines of the Declaration of Helsinki and approved by the local ethics committee of the Endocrinology Research Centre, Ministry of Health of Russia, Moscow, Russia (protocol No. 17 and date of approval 27 September 2017). Informed consent was obtained from all subjects involved in the study.

### Clinical samples and genomic DNA isolation

The recruitment period for this study is 18.07.2019–22.03.2022. Serum samples were collected from patients and stored at −80°C. Venous blood samples were collected from both patients and their parents. Genomic DNA was extracted from total peripheral human blood using the QIAamp DNA Blood Midi Kit (Qiagen, Hilden, Germany) in accordance with the manufacturer’s instructions.

### Detection of autoantibodies in serum samples by ELISA kits and microarray-based assay

All patients’ serum samples were measured by ELISA to detect autoantibodies (autoAbs) to P450c21 (21-hydroxylase, 21-OH, BioVendor Laboratory Medicine, Brno, Czech Republic), thyroperoxidase (TPO) (Abbott Laboratories, Chicago, IL, USA), and thyroglobulin (TG) (Roche Diagnostics, Basel, Switzerland); tyrosine phosphatase (IA2), islet cell antigens (ICA), zinc transporter 8 (Zn8) (all–from Medipan, Berlin, Germany); glutamic acid decarboxylase (GAD) (Biomerica, Irvine, CA, USA); insulin (IAA), parietal cells of the stomach (ATP4), Castle intrinsic factor (GIF) (all–from Orgentec, Mainz, Germany). Serum sample of Patient C was additionally tested for antibodies (Abs) against gliadin (GLD) (Vector-Best, Novosibirsk, Russia) and tissue transglutaminase (TGM2) (Orgentec, Mainz, Germany).

Serum samples of patients were assayed using a hydrogel microarray with immobilized antigen for detection of autoAbs to interleukin-22 (IL-22), omega and alpha-2-a interferons (IFN-ω and IFN-α-2a) and organ-specific autoAbs to 21-OH, GAD, IA2, ICA, TG, and TPO. The microarrays were manufactured as described previously [16]. Each antigen was immobilized in 4 repetitions to increase the reproducibility of the assay results. Patient serum samples were diluted 1:100 (100 mM Tris-HCl buffer with 0.1% Triton X-100) and applied to the microarray (100 μl). After overnight incubation at 37°C, intermediate washing (PBS with 0.1% Tween 20, 20 min), rinsing, and drying, the microarrays were developed with fluorescently labeled anti-species Abs. As detecting Abs, we used F(ab’)2-Goat anti-Human IgG Fc gamma Secondary Ab (Cat #31163, Invitrogen, Carlsbad, CA, USA) labeled with Cy5 cyanine dye. After incubation for 30 min at 37°C, the microarrays were washed (PBS with 0.1% Tween 20, 30 min), rinsed and dried by centrifugation. Registration of fluorescent images of microarrays and calculation of fluorescent signals were performed using a microarray analyzer and software developed at the Engelhardt Institute of Molecular Biology, Moscow. Interpretation of the results of analysis on a microarray with the determination of the presence/absence of autoAbs in blood serum were performed as described previously [16,17].

### Exome sequencing and genetic examination

DNA Libraries were prepared from 500 ng of genomic DNA using the MGIEasy Universal DNA Library Prep Set (MGI Tech, Shenzhen, China) according to the manufacturer’s protocol. DNA fragmentation was performed via ultrasonication using Covaris S-220 (Covaris, Inc., Woburn, MA, USA) with an average fragment length of 250 bp. Whole-exome enrichment of DNA library pools was carried out according to a previously described protocol [18] using the SureSelect Human All Exon v7 probes (Agilent Technologies, Santa Clara, CA, USA). The concentrations of DNA libraries were measured using Qubit Flex (Life Technologies, Carlsbad, CA, USA) with the dsDNA HS Assay Kit (Invitrogen, Waltham, MA, USA) following the manufacturer’s protocol. The quality of the prepared libraries was assessed using Bioanalyzer 2100 with the High Sensitivity DNA kit (Agilent Technologies, Santa Clara, CA, USA) according to the manufacturer’s instructions.

The enriched library pools were further circularized and sequenced via paired end sequencing using DNBSEQ-G400 with the DNBSEQ-G400RS High-throughput Sequencing Set PE100 following the manufacturer’s instructions (MGI Tech, Shenzhen, China), with an average coverage of 100×. Fastq files were generated using the basecallLite software (ver. 1.0.7.84) from the manufacturer (MGI Tech, Shenzhen, China).

Bioinformatics analysis of sequencing data was performed through a series of steps. The quality control of the obtained paired fastq files was executed using FastQC v0.11.9 [19]. Based on the quality metrics, fastq files were trimmed using BBDuk by BBMap v38.96 [20]. Trimming data were aligned to the indexed reference genome GRCh37 using bwa-mem2 v2.2.1 [21]. SAM files were converted into bam files and sorted using SAMtools v1.10 to check the percentage of the aligned reads [22]. The duplicates in the obtained bam files were marked using Picard MarkDuplicates v2.22.4 [23] and excluded from further analysis.

Quality control analysis was conducted on marked bam files using the Agilent all-exon v7 target file ‘regions.bed’. For samples that passed quality control (with a target coverage width of 10× ≥ 95%), single-nucleotide variants and indels were called using the bcftools mpileup software v1.9 [24], and vcf files were obtained for each sample.

Alignment quality was assessed on a bam file with labeled duplicates, utilizing a BED file containing information on targeting regions. After variant calling, vcf files were normalized with vt normalize v0.5772 [25] and filtered based on the target regions expanded by ± 100 base pairs flanking each end. Variant calling data were annotated using the InterVar software [26]. The clinical significance of nucleotide sequence variants was evaluated based on the ACMG criteria [27], with variants having a read depth of 14× or higher exclusively selected for consideration.

Genes from the HPO ‘Autoimmunity’ panel (HP:0002960) [28], excluding HLA genes, and our custom panel based on literature data (S1 Table) were analyzed. A group of 637 healthy volunteers, who had undergone whole-exome sequencing in a previous study [29], was utilized as controls. Before performing the transmission disequilibrium test (TDT), 1790 variants were removed from the vcf file due to genotyping quality less than 95%. We conducted TDT and principal component analysis (PCA) utilizing plink v1.90b6.24 software [30] to determine the genetic distance of the samples. Due to the small sample size, corrected P values were not reported as all variants exhibited P > 0.05 in the ‘.tdt.adjusted‘ file. To evaluate relatedness, identity by descent (IBD) was employed as an additional control, along with default parameters for Runs of Homozygosity (ROH). Graphical data depiction was executed utilizing R version 4.2.3 (R Foundation for Statistical Computing, Vienna, Austria). Charts of gene enrichment analysis following DisGeNET categories were created using Metascape v3.5.20230101 [31], and UpSet plot was generated using the SRPLOT online tool (http://www.bioinformatics.com.cn/srplot (accessed on 18 November 2023)).

### High-resolution HLA typing

The preparation of amplicon libraries for HLA high-resolution genotyping was conducted using the HLA Expert kit (DNA Technology LLC, Moscow, Russia), following the manufacturer’s protocol (the kit was approved by Russian Federal Service for Surveillance in Healthcare–Roszdravnadzor). The process consisted of several steps. Firstly, a qPCR for human gene with no pseudogenes and presented in a single copy was performed in the initial stage. This was necessary for estimating the concentration and detecting inhibitors in the genomic DNA sample. The results were utilized for normalizing the DNA amount during in the subsequent step. The second stage involved a multiplex PCR targeting most variable exons (exons 2, 3, 4 for the HLA class I and 2, 3 for the HLA class II). Primers were designed using conservative regions of gene introns flanking the exons. To prevent an imbalance in nucleotide content during sequencing, several primers with a one-nucleotide shift were employed. The third stage encompassed ligation of the adapters containing Illumina i5 and i7 indexes. The fourth stage included an additional routine PCR (6 cycles) with the p5 and p7 primers. Purification with magnetic beads (SPRI type) was carried out after each stage. Quality control of the libraries was conducted using agarose gel electrophoresis, and the concentration was measured using the Qubit 3 fluorometer with the Qubit dsDNA HS Assay kit (ThermoFisher Scientific, USA).

Sequencing was executed using the Illumina MiSeq platform (Illumina, San Diego, CA, USA) with the MiSeq Reagent Kit v3 (600-cycle), according to the manufacturer’s protocol.

Fastq files were analyzed with HLA-Expert software (DNA Technology LLC, Moscow, Russia) following the manufacturer’s instructions. The obtained exon sequences were aligned to the MHC sequences IMGT/HLA v3.41.0 [32].

Basic quality control metrics included: 1). Setting a quality threshold for reads, with low-quality reads either trimmed or discarded. 2). Determining the lowest absolute and relative coverage for each position. 3). Identifying the highest number of differences (insertions, substitutions, deletions) from the group average for each read. 4). Specifying a maximum relative position error, where the number of differences (insertions, substitutions, deletions) from the consensus sequence in each position should not exceed the specified threshold. 5). Calculating the highest average error per read for a group. 6). Establishing the lowest number of reads in groups for each exon (class I– 2, 3, 4 exons, class II– 2, 3 exons). 7). Ensuring that allelic imbalance does not exceed a given threshold, which involves considering the ratio of the read number for the exons from each allele and the sum of these ratios. 8). Checking for the presence of phantom (cross-mapping) and chimeric sequences. 9). Calculating the percentage of combined, clustered, and used for typing reads computed for each sample.

Haplotype frequencies were estimated by Arlequin version 3.5.2.2 [33] using the expectation-maximum algorithm. Frequencies were determined for the class I loci, class II loci, five loci (A, B, C, DRB1, DQB1) and six loci (A, B, C, DRB1, DQB1, DPB1). A control group was established using a bone marrow donor registry of 1999 individuals, with 647 individuals from the same cohort specifically used for the *HLA-DQA1* locus.

### Sanger sequencing

The following SNPs were verified by Sanger sequencing: rs2476601 in the *PTPN22* gene, rs731236 and rs7975232 in the *VDR* gene, rs72650691 and rs1183715710 in the *CLEC16A* gene, rs538912281 in the *FOXE1* gene, and rs758401262 in the *ABCA7* gene. Sanger sequencing was performed using a 3730xl Genetic Analyzer (Applied Biosystems, Foster City, CA, USA). Nucleotide sequence alignment was performed using the Chromas Lite 2.1 program (Technelysium Pty Ltd, Brisbane, Australia). A comparative analysis of DNA sequences and polymorphisms was conducted using the Unipro UGENE software [34], with reference to corresponding regions of the *PTPN22*, *VDR*, *CLEC16A*, *FOXE1* and *ABCA7* genes from the GenBank database.

## Results

### Detection of autoantibodies in serum samples of patients

The results of testing patient serum samples using ELISA kits and a microarray-based assay are shown in Tables 2 and S2 and S1 Fig. Abs against 21-OH were found in all patients through ELISA testing (Patient A, 51.672 U/mL; Patient B, 40.728 U/mL, Patient C: 53.779 U/mL, normal <0.4 U/mL, S2 Table), verified by qualitative microarray analysis. The immunologic test results confirmed the autoimmune etiology of primary adrenal insufficiency in all three patients. Furthermore, Patient A showed elevated Abs to IA2, measuring 27.6 U/mL (normal range: 0–10 U/mL). Patients A and B had elevated TPO Abs, while their TG Abs were within the normal range according to both methods. In contrast, Patient C’s insulin Abs were notably increased (18.4 U/mL), but their Abs to IA2, Zn8, ICA, and GAD were all within standard reference values. Furthermore, Abs to GLD and tissue TGM2 were not detected. None of the patients had Abs to type I IFN and IL-22 by microarray analysis, which made it possible to exclude the diagnosis of APS-1, for which these markers are highly specific [16,17]. The latter was especially important for Patient C, who, in addition to AAI, was diagnosed with esophageal candidiasis.

**Table 2:** The presence of autoantibodies in patient serum samples was assessed using microarray-based and enzyme-linked immunosorbent immunoassays.

Autoantibodies	Patient A	Patient B	Patient C
Anti-21OH ELISA/Microarray	+/+	+/+	+/+
Anti-TPO ELISA/Microarray	+/+	+/+	-/-
Anti-Tg ELISA/Microarray	-/-	-/-	-/-
Anti-IA2 ELISA/Microarray	+/-	-/-	-/-
Anti-ICA ELISA/Microarray	-/-	-/-	-/-
Anti-GAD ELISA/Microarray	-/-	-/-	-/-
Anti-Zn8 ELISA	-	-	-
Anti-IAA ELISA	-	-	+
Anti-ATP4 ELISA	-	-	-
Anti-GIF ELISA	-	-	-
Anti-GLD ELISA	not tested	not tested	-
Anti-TGM2 ELISA	-	-	-
Anti-IFN-ω Microarray	-	-	-
Anti-IFN-α Microarray	-	-	-

Notes: 21-OH, 21 hydroxylase; TPO, thyroperoxidase; TG, thyroglobulin; IA2, tyrosine phosphatase; ICA, islets of Langerhans cells; GAD, glutamate decarboxylase; Zn8, zinc transporter; IAA, insulin; ATP4, parietal cells of the stomach; GIF, Castle intrinsic factor; GLD, gliadin; TGM2, tissue transglutaminase; IFN-ω, omega interferon; IFN-α, alpha interferon; IL-22, interleukin 22.

A synthesis of the clinical and laboratory data pertaining to the patients reveals the presence of manifest APS-2 in patients A and B, and latent APS-2 in patient C (considering the presence of antibodies to insulin). In all cases, the disease manifested at a relatively young age.

### Characterization of the exome data

Tables 3 and S3 present the primary quality control metrics for whole-exome sequencing of patient samples, including the sex chromosome karyotype and outcomes of the *SRY* gene analysis, which matched the information provided in the patient medical records. All nine exome datasets met high-quality standards for further analysis.

**Table 3:** Quality control metrics for exome sequencing of samples.

Metrics	Mean	Min	Max
Single reads per sample	102,044,084	83,932,144	154,800,134
Estimated library size	162,054,959	106,209,935	226,432,803
Duplicates	14.89	8.70	20.00
On-target bases	88.1%	87.2%	88.7%
Mean target coverage	108.93	90.30	159.50
Median target coverage	102.33	78.00	154.00
Width 10×	95.80%	93.10%	97.00%
Width 20×	93.82%	90.20%	96.40%
Width 30×	91.14%	87.80%	95.70%

**Fig 2:**
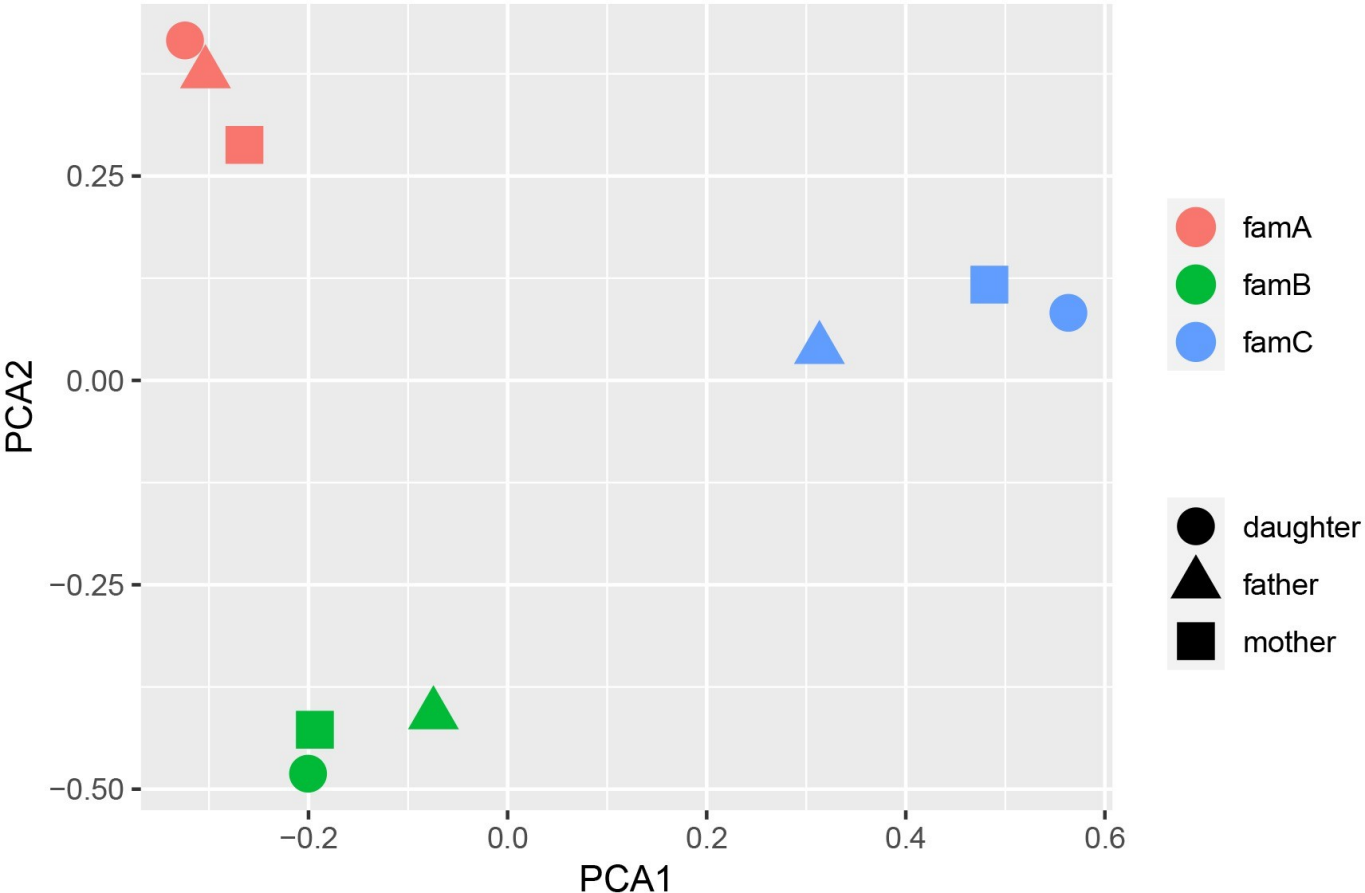
Principal Component Analysis (PCA) of the genotypes for three families reveals a distinct clustering of subjects from Families 1, 2, and 3 on the resulting plot, with the first primary component on the x-axis and the second primary component on the y-axis. Each individual is color-coded according to their respective family.

The overall genotyping quality of the samples was 99.75%. PCA data showed a pronounced genetic distance between families (Fig 2). A total of 2497 variants in sex chromosomes were excluded during PCA, and 155,188 variants in autosomes were present in the sample.

Based on the IBD analysis, the daughters inherited approximately 50% of the variants from each parent (refer to S4 Table). Additionally, 17.26% of the variants present in the parents of the Family A were found to overlap. The ROH analysis identified a single region on Chromosome 1 of Proband B, spanning 1083.817 kb from genomic position 45,206,495 to 46,290,311, with a SNP distribution density of 10.035 and 99.1% of homozygous variants. This region aligned with the paternal range (45,163,647–46,487,552) and did not contain any pathognomonic genes. Notably, the proband’s mother exhibited a higher proportion of heterozygous variants in this region than allowed for ROH.

### Analysis of genetic variants

Table 4 summarizes APS-specific genetic variants that we have detected and that were previously described [35,36].

**Table 4:** Variants described in the literature found in patients (coverage of 14× and above for all examined samples).

Variant	Patient A (VAF)	Patient B (VAF)	Patient C (VAF)	P-value for TDT
*CTLA4* (NM_005214.5):c.49A>G (p.Thr17Ala) rs231775	+/+ (1)	+/- (0.482456)	+/- (0.492958)	0.0455
*PTPN22* (NM_015967.7):c.1858T>C (p.Trp620Arg) rs2476601	+/+ (1)	+/+ (1)	+/+ (1)	NA*
*NFATC1* (NM_001278669.2):c.2251T>G (p.Cys751Gly) rs754093	+/- (0.504505)	+/- (0.422535)	-/-	0.5637
*GPR174* (NM_032553.3):c.484T>C (p.Ser162Pro) rs3827440	+/- (0.510526)	+/- (0.465347)	+/+ (0.996855)	0.3173
*VDR* (NM_000376.3):c.1025-49G>T rs7975232	-/-	+/+ (1)	+/+ (1)	0.3173
*VDR* (NM_000376.3):c.1056T>C (p.Ile352 =) rs731236	-/-	+/- (0.503759)	+/- (0.466165)	1

Designations: TDT, transmission disequilibrium test. Notes: *all family members possess the variant in a homozygous state, +/+ homozygous for alternative allele, +/- heterozygous, -/- homozygous for reference allele.

The only variants found on the same chromosome were rs7975232 and rs731236, with a likelihood odds ratio of 32.85, as determined in the study by Maciejewski A et al. [37].

No clinically significant gene variants, including those in the *AIRE* gene, were identified from the HPO ‘Autoimmunity’ panel HP:0002960 [28] (S5 Table). Detailed results of the interpretation of nucleotide sequence variants are presented in S6 Table, which is based on our custom panel utilizing literature data. S7 Table showcases variants marked as pathogenic by at least one Clinvar user. Given the high frequency of most variants within the healthy population, we recommend interpreting them as ‘risk alleles‘. A heterozygous variant of uncertain clinical significance (VUS) *FOXE1*(NM_004473.4):c.743C>G (p.Ala248Gly) in patient B was inherited from the mother and has been identified as a risk factor for thyroid carcinoma [38]. The *FOXE1* gene is also associated with the development of autoimmune thyroiditis [39,40]. The paternally transmitted heterozygous pathogenic variant *MCCC2*(NM_022132.5):c.1574+1G>A from patient B is associated with 3-Methylcrotonyl-CoA carboxylase 2 deficiency. Since this disease follows Mendelian autosomal recessive inheritance, no additional phenotype is expected, and we did not find a second causative allele. Hence, the variant was not incorporated into S7 Table. For an identical reason, we did not include carriage of the pathogenic variant *COL7A1*(NM_000094.4):c.682+1G>A in patient C, inherited from her mother and associated with recessive dystrophic epidermolysis bullosa, as well as the likely pathogenic variant *HFE*(NM_000410.4):c.187C>G (p.His63Asp associated with hemochromatosis, type 1, which she inherited from her father. Patient C has the repeatedly described in the literature *BRCA1*(NM_007294.4):c.5074G>C (p.Asp1692His), which is consistent with the genealogical history: her maternal grandmother was diagnosed with breast cancer.

Subsequently, we investigated variants that have not been previously reported in the clinic (S6 Table) and were classified as not benign and likely benign based on the criteria of the American College of Medical Genetics and Genomics (ACMG) [27].

Patient A inherited VUS in the *CLEC16A* gene (NM_015226.3):c.3097G>A (p.Ala1033Thr) from their father, with both being heterozygous. No homozygotes have been reported in the Genome Aggregation Database (gnomAD), and no syndrome is associated with this variant in the Online Mendelian Inheritance in Man (OMIM) database. Despite in silico predictions of benignity, the variant is considered a VUS due to the unknown inheritance type (it may be digenic dominant) or a risk allele. The *CLEC16A* gene has been described in broad association with autoimmune diseases, among them autoimmune thyroiditis and AAI [41].

Patient B has a heterozygous maternally transmitted VUS *CLEC16A*(NM_015226.3):c.2083G>A (p.Asp695Asn) and VUS/risk allele *ABCA7*(NM_019112.4):c.3187G>A (p.Glu1063Lys), the former variant is not present in gnomAD. The *ABCA7* gene encodes a protein responsible for phagocytosis [42]. Some evidence suggests that individuals with schizophrenia may have abnormal autoimmune responses and higher levels of pro-inflammatory cytokines in their blood [43]. This finding could potentially extend to other immune disorders as well.

Patient C did not exhibit any new clinically significant variants from the gene panel.

**Fig 3:**
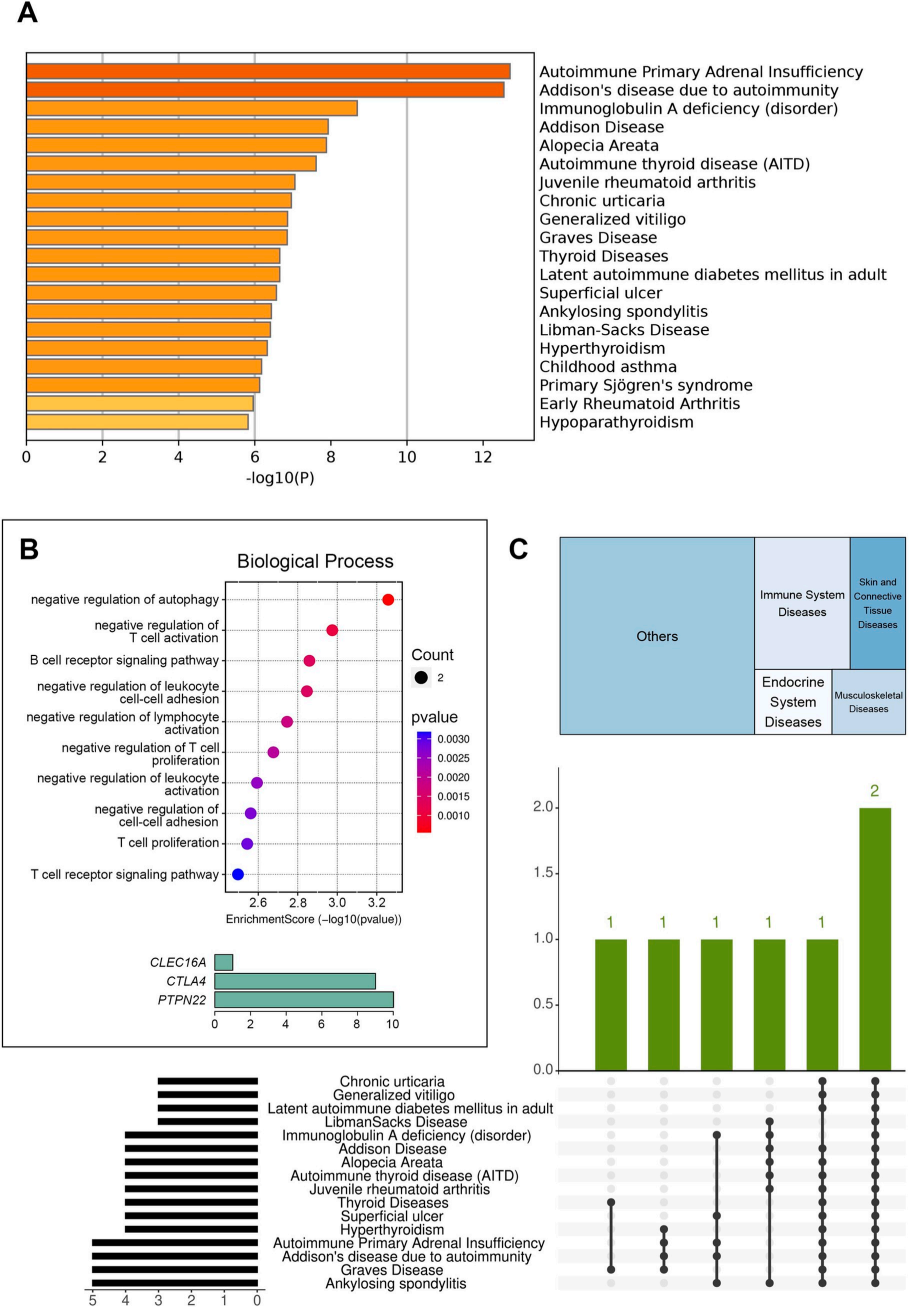
A summary of enrichment analysis in DisGeNET for patient B, generated by Metascape (**A**); performed using SRplot: A bar plot for representative terms of gene ontology enrichment analysis and a frequency histogram showing the number of genes for the top-10 terms (**B**); a treemap representing disease class and an UpSet plot showing how disease-causing genes intersect (**C**).

Given that Patient B had the highest number of variants in genes associated with autoimmune diseases among all probands, we conducted gene enrichment analysis for her using Metascape and SRplot (Fig 3).

### Overview of HLA system risk and protective alleles/haplotypes in studied families and controls

The results of HLA typing of class II loci are presented in Tables 5 and S8. In Family A, the daughter had DRB1*03:*01*:*01*:*01G*, DQA1*05:*01*:*01*:*01G* and DQB1*02:*01*:*01*:*01G* alleles, characteristic of APS-4 [35], inherited from the father. The frequency of this haplotype in the healthy Russian population is 6.4912%. Probands from families B and C possessed alleles related to APS types 2, 3, and 4. Notably, the alleles DQA1*03:*01*:*01G* and DQB1*03:*02*:*01G* are characteristic of APS-2 and APS-3. The DRB1*04:04~DQA1*03:01~DQB1*03:02 haplotype related to APS-2 has an incidence rate of 1.8098% among healthy volunteers. Meanwhile, the DRB1*04:01~DQA1*03:01~DQB1*03:01~DQB1*03:*02* haplotype characteristic for APS-3 has occurrence rate of 1.9347%.

**Table 5:** Genotyping of HLA loci (DRB1, DQA1, DQB1, DPB1) at G-group resolution in three families.

Family	Member	HLA-DRB1*		HLA-DQA1*		HLA-DQB1*	
**A**	Mother	04:04:01G	12:01:01G	03:01:01G	05:01:01G	03:01:01G	03:02:01G
	Father	03:01:01G	11:01:01G	05:01:01G	05:01:01G	02:01:01G	03:01:01G
	Daughter	03:01:01G	12:01:01G	05:01:01G	05:01:01G	02:01:01G	03:01:01G
**B**	Mother	04:03:01G	11:01:01G	03:01:01G	05:01:01G	03:01:01G	03:02:01G
	Father	03:01:01G	15:02:01G	01:03:01G	05:01:01G	02:01:01G	06:01:01G
	Daughter	03:01:01G	04:03:01G	03:01:01G	05:01:01G	02:01:01G	03:02:01G
**C**	Mother	03:01:01G	13:01:01G	01:03:01G	05:01:01G	02:01:01G	06:03:01G
	Father	04:03:01G	15:01:01G	01:02:01G	03:01:01G	03:02:01G	06:02:01G
	Daughter	03:01:01G	04:03:01G	03:01:01G	05:01:01G	02:01:01G	03:02:01G

We analyzed the HLA haplotype frequencies of both our patients and healthy volunteers from the bone marrow donor registry, as shown in S9 Table. Of the six proband haplotypes resulting from a combination of alleles from seven genes, four were not encountered in the control sample and had a frequency of less than 0.0624%. The remaining two haplotypes have frequencies of 0.2% and 2%, respectively, and are common in the population. Following partitioning and analysis into separate classes, namely class I with three genes and class II with four genes, class I showed a total of six unique haplotypes with four occurrences (frequencies of 2%, 4%, 0.5%, and 0.025%), while two occurrences with a frequency less than 0.003% were not found in the control sample. In class II, the frequencies were 3%, 6%, 0.2%, 0.3%, and 0.9%.

## Discussion

Autoimmune diseases, including autoimmune endocrinopathies and APS, are still under investigation for their genetic causes. This work highlights the importance of genetic verification, which can predict the disease from birth, in contrast to immunologic and biochemical studies. The example of patients with autoimmune adrenal insufficiency, a life-threatening immune-mediated disease, demonstrates possible solutions to this problem.

Our study involved genetic testing not only for the patients with AAI but also their family members (specifically the parents). This design was chosen to ensure accurate interpretation of the genetic findings. Therefore, it is imperative to analyze the clinical characteristics and genetic information of the patient and their family members to assess phenotype-genotype correlations. Identified pathogenic variants or variants of uncertain significance in parents may contribute to the subclinical course of the disease. Conversely, the absence of pathology in parents with pathogenic mutations may be due to protective variants in other genes or environmental factors. The patient’s disease development may result from a complex interplay of various genetic factors.

All patients had serum autoAbs against 21-OH, confirming the significance of this marker for the diagnosis of AAI [44]. In the analyzed group, all individuals had AIRE gene variants that were either benign or likely benign (S5 Table). Based on the clinical data and results indicating an absence of Abs against IFN-α and -ω, as well as IL-22, APS-1 has been ruled out. This determination was particularly crucial for Patient C, who had been diagnosed with esophageal candidiasis in addition to AAI. However, recent evidence highlights heterozygous or dominant-negative mutations causing only partial AIRE gene function loss. Such defects could contribute to the development of varied autoimmune diseases in the affected person, but with a delayed onset, mild course, and not necessarily manifested in multiple forms [45]. Carriage of Abs without apparent disease has also been reported [36].

The search for new genetic predictors of APS in the Russian population is constrained by two studies conducted by the authors of the present study [41,46]. In the first study, a complex heterozygous mutation in the *VTCN1* gene (c.[-21C>A];[-66C>T]) was identified in monozygotic twins with AAI as part of APS-2. It was determined that the parents of patients, who were deemed healthy, were heterozygous carriers of variants of this gene (in the mother, c.-21C>A; in the father, c.-66C>T). In the second study, the results of an examination of a patient with an atypical course of APS-2 manifesting in childhood and her parents suggest that the *PTPN22*, *PJA2*, *CASR*, and *DDX60L* genes, which are involved in the functioning of the endocrine and immune systems, may serve as genetic predictors of APS, particularly exhibiting a dominant-negative effect. In examining a patient with an atypical course of APS-1 manifesting in adulthood, a novel combination of heterozygous mutations in the *AIRE* gene (c.769C > T and c.821delG) was identified.

Significant associations between variants and diseases as defined by genome-wide association studies (GWAS) usually have a frequency of over 3% in gnomAD. This means that they are considered to be benign based on the ACMG criteria. However, the rarity of AAI in Russia makes it unfeasible to collect DNA samples from thousands of patients required to perform GWAS to determine the significance of certain variants for pathogenesis. For instance, primary adrenal insufficiency has a global prevalence rate of 115 patients per 1 million people [44]. For Russia, a country with a population exceeding 140 million people, conducting GWAS studies is feasible. Nevertheless, the absence of a unified register of patients with AAI in the Russian Federation and the fragmented information on patients significantly complicates the process [47].

In the present study, we identified established mutations in the *CTLA4*, *PTPN22*, *NFATC1*, *GPR174*, and *VDR* genes, which are linked to endocrine autoimmunity [48–52], that were inherited from their parents in patients with this condition. Patients with APS-2, in contrast to the patient with AAI only, each inherited a distinct VUS in the *CLEC16A* gene: (NM_015226.3):c.3097G>A (p.Ala1033Thr) in Patient A and (NM_015226.3):c.2083G>A (p.Asp695Asn) in Patient B. These variants have not been previously described. *CLEC16A* genetic variations have been associated with various autoimmune diseases, including multiple sclerosis, type 1 diabetes, Crohn’s disease, AAI, rheumatoid arthritis, and juvenile idiopathic arthritis [53].

In this study, a whole-exome analysis was conducted instead of a whole-genome analysis. It is important to note that approximately 90% of the variants identified through GWAS are located in noncoding regions, including intronic and intergenic regions. Chromosomal crossovers among patients with various autoimmune diseases are undeniable, making it challenging to distinguish between different nosologies at the genomic level [54]. In the presented case, Patient A manifests variants in the *AMPD1*, *PRSS1*, *STOX1*, *MBL2* and *SAA1*, genes. Further, Patient B displays putative pathogenic variants in the *KLKB1*, *MCCC2*, *PRSS1*, *SAA1*, *PTPRJ*, *ABCC6*, *SERPINA7*, *IL4R*, *BCHE* genes; while Patient C exhibits such variants in the *KLKB1*, *FGFR4*, *ASAH1*, *STOX1*, *SAA1*, *TENM4*, *ABCC6*, *PKD1*, *COL7A1*, *HFE*, *BRCA1* and *CST3* genes (as partially shown in S6 Table). Associations have been reported between nonendocrine autoimmune pathologies, such as rheumatoid arthritis, systemic lupus erythematosus, multiple sclerosis, and autoimmune myocarditis, and most of the genes listed. *SAA1* and *ABCC6* gene variants were present in all patients of the study. However, current evidence does not support linking these variants to autoimmune endocrinopathies, as they can also occur in patients without autoimmune diseases [55].

Comparable challenges arise when attempting to associate a single variant to pathology as they are often situated within the haplotype [56]. Additionally, 22% of loci connected with APS display heterogeneity in phenotype [57]. Haplotypes presumed to be related to a certain disease are found in a healthy population with high frequency. The occurrence of the disease cannot be wholly attributed to the haplotype allele combination since there exists significant linkage disequilibrium between genes present in haplotypes. Thus, if only one of the genes contributes to disease causality, the alleles of the other genes may not have any pathogenetic significance. *HLA-DRB1* could be considered the pivotal gene in this case, based on its connection with autoimmune diseases, in tandem with the DRB1*04 allele group. The *HLA-DQB1* and *HLA-DQA1* alleles, which also figure in other haplogroups, further support this claim.

The proband of Family A was characterized by alleles DRB1*03:*01*:*01*:*01G*, DQA1*05:*01*:*01*:*01G* and DQB1*02:*01*:*01*:*01G* associated with APS-4. However, the significant frequency of this allele in the population, at 6.4912%, does not establish a clear association with the disease. We believe that HLA typing outcomes may be influenced by the presence of rare Family A alleles in European Russia. Additionally, the *HLA-A* and *HLA-DPB1* genes pose challenges for haplotype analysis due to their distance from other MHC genes and minimal linkage disequilibrium. Probands from Families B and C possessed alleles related to APS types 2, 3, and 4. Specifically, alleles DQA1*03:*01*:*01G* and DQB1*03:*02*:*01G* are characteristic of APS-2 and APS-3, respectively. Nevertheless, the frequencies of these haplotypes found in both Patients B and C are much more prevalent in the general population than the disease associated with them.

Thus, in routine clinical diagnosis, WES and even HLA typing are not exhaustive in establishing the type of APS and APS itself (in the case of types 2, 3 and 4). In the future, to discover genetic predictors of autoimmune endocrinopathies, the entire genome of patients and their family members should be analyzed, including non-coding regions such as intronic and intergenic regions, to find clinically significant variants. Furthermore, this study should be conducted on a much larger patient sample.

Nonetheless, whole-genome sequencing (WGS) and CytoScan HD array analysis of the APS-2 patient and his father, along with a thorough investigation that included important immunoregulatory genes (i.e., *CTLA-4*, *PTPN22*, *STAT4*), did not identify any pathogenic variants or copy number alterations linked to the phenotype. While WGS is a useful tool, it might not always capture all pertinent genetic variables. This finding emphasizes the complexity and potential limitations of existing genomic screening approaches in detecting APS-2 [58].

## Conclusions

Our work underlines the necessity of adopting a multidisciplinary strategy towards APS, combining genetic data with immunological markers and clinical observations for better risk assessment. High-resolution HLA typing and a trio-based WES examination, however, did not yield any reliable indicators of autoimmune endocrinopathies. Future studies involving larger patient cohorts and more advanced sequencing methods, such as WGS with a focus on non-coding regions, may uncover novel genetic predictors and deepen our understanding of the etiology of autoimmune diseases. This strategy could also facilitate the development of personalized treatment and monitoring plans for individuals with APS.

## Supporting information

S1 Fig Fluorescence images of the microarray and medians of normalized signals from the microarray elements after assaying the serum samples from patients. (PDF)

S1 Table A list of genes linked to autoimmune diseases, excluding HLA, currently under investigation. (XLSX)

S2 Table Detection of Auto-antibodies by ELISA kits. (XLSX)

S3 Table Picard CollectHsMetrics output with sex verification and analysis of heterozygosity. (XLSX)

S4 Table Identity by descent (IBD) of members of three families. (XLSX)

S5 Table Genetic variants detected by using a HPO panel (Family A, Family B, Family C). (XLSX)

S6 Table Genetic variants detected using a custom panel (Family A, Family B, Family C). (XLSX)

S7 Table Variants assigned Clinvar pathogenic clinical significance. (XLSX)

S8 Table Results of high-resolution HLA typing. (XLSX)

S9 Table Haplotype frequencies for family members. (XLSX)
