# Supplementary material for: Trio-based exome sequencing and high-resolution HLA typing in families of patients with autoimmune adrenal insufficiency and autoimmune polyglandular syndrome
Source: PLoS One. 2024 Oct 18;19(10):e0312335. doi: 10.1371/journal.pone.0312335 (PMC11488712; doi:10.1371/journal.pone.0312335)
Supplement: S1 Fig — (PDF) [file pone.0312335.s001.pdf]

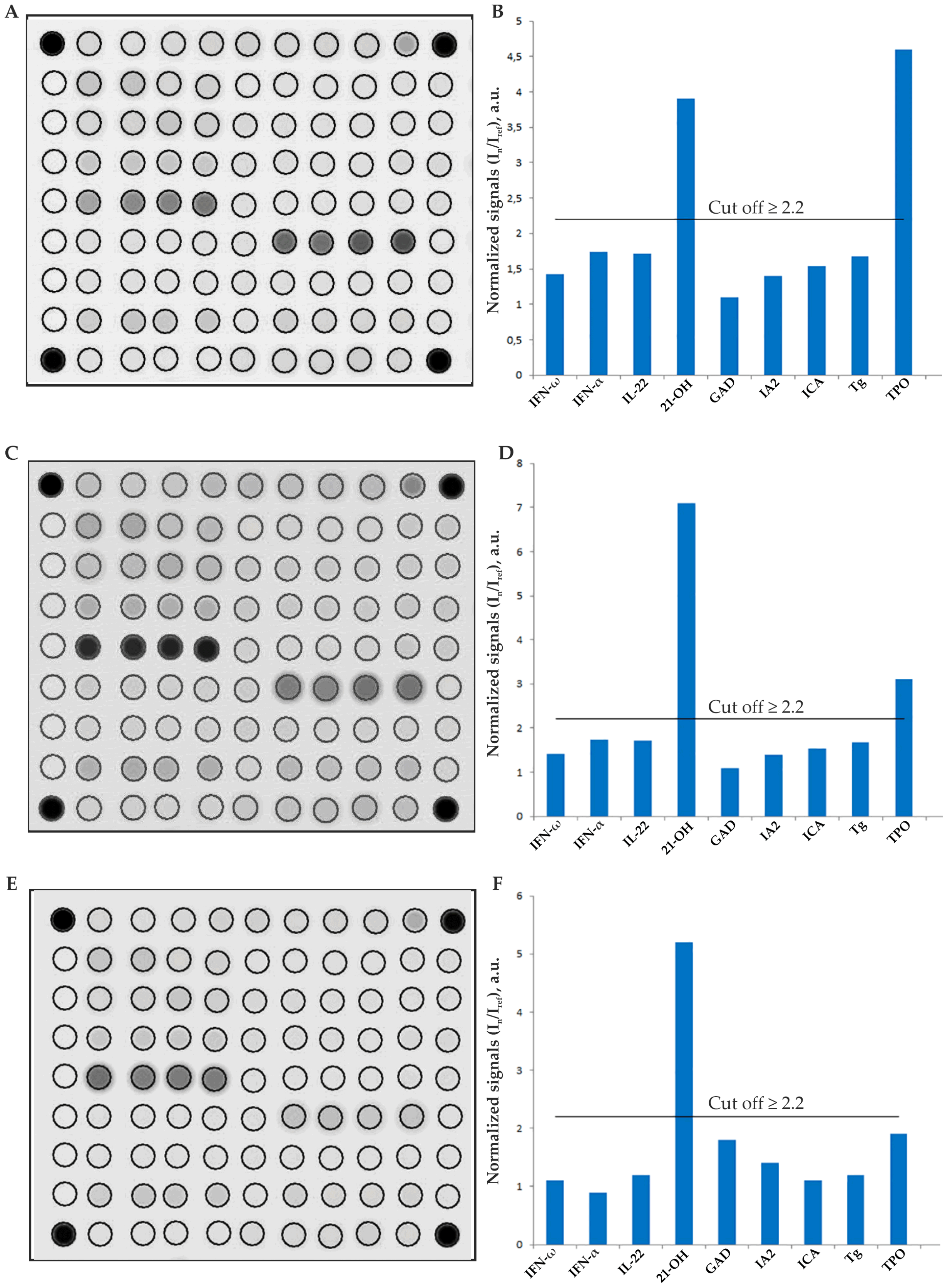

Fig S1. Fluorescence images of the microarray and medians of normalized signals from the microarray elements after assaying the serum sample from Patient A (**A,B**); Patient B (**C,D**); Patient C (**E,F**). Interpretation of results was performed as previously described (Savvateeva et al., 2021; Ref. #16). Designations: IFN-ω – intierferon omega; IFN-α-2a – interferon-alpha-2a; IL-22 – interleukin 22; 21-OH – steroid 21 hydroxylase; GAD – glutamic acid decarboxylase 65 kDa; IA2 – tyrosine phosphatase-like autoantigen; ICA – islet cell autoantigen 1; TPO – thyroid peroxidase; TG – thyroglobulin.
